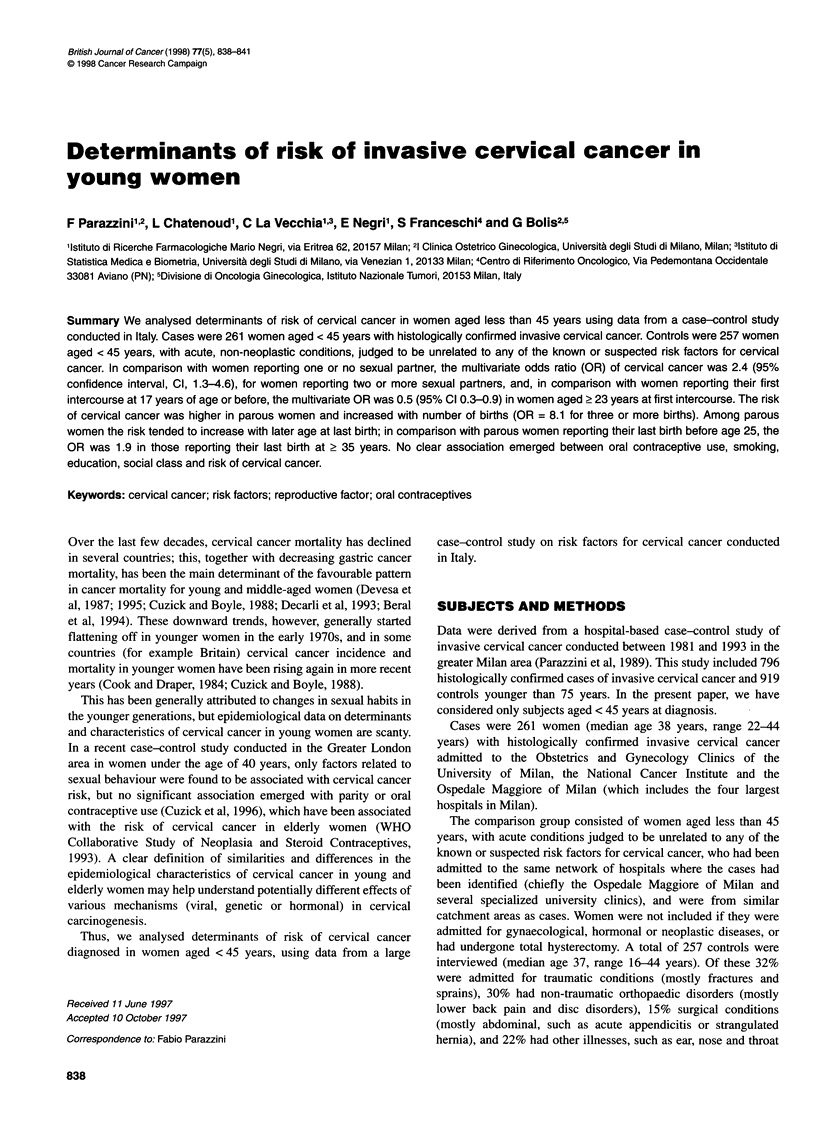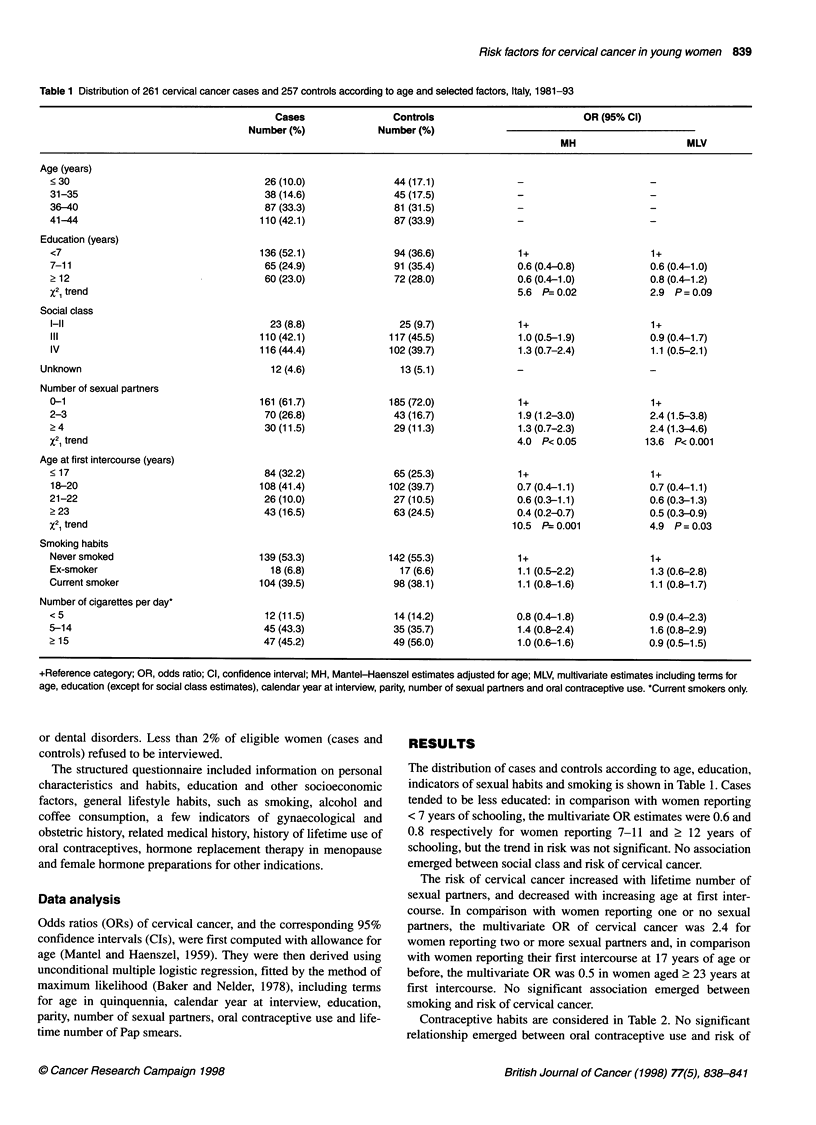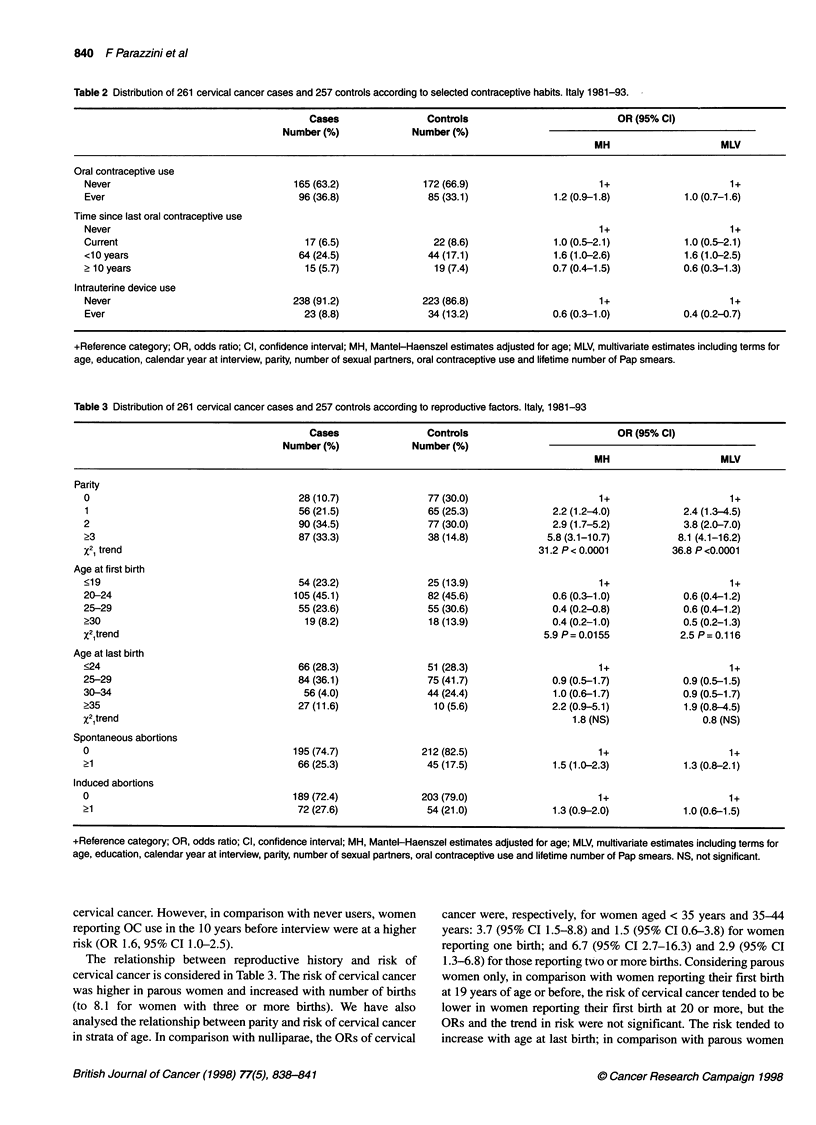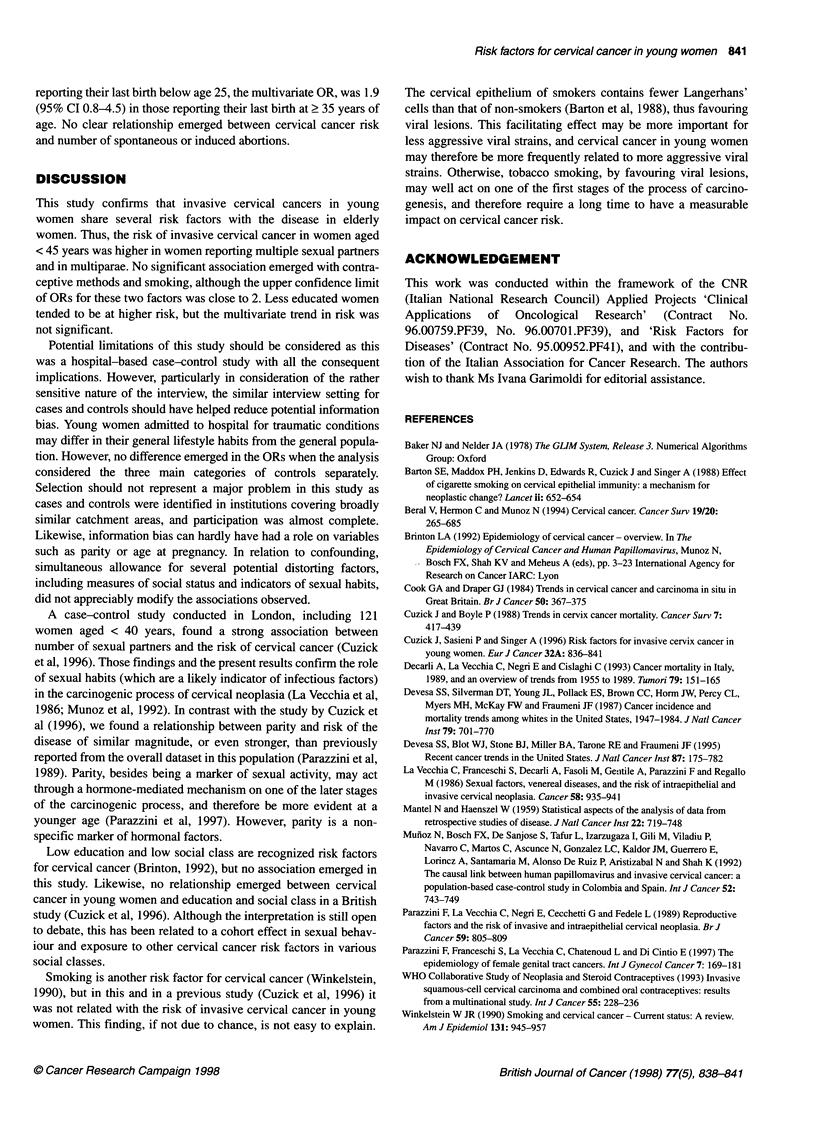# Determinants of risk of invasive cervical cancer in young women.

**DOI:** 10.1038/bjc.1998.136

**Published:** 1998-03

**Authors:** F. Parazzini, L. Chatenoud, C. La Vecchia, E. Negri, S. Franceschi, G. Bolis

**Affiliations:** Istituto di Ricerche Farmacologiche Mario Negri, I Clinica Ostetrico Ginecologica, UniversitÃ degli Studi di Milano, Milan, Italy.

## Abstract

We analysed determinants of risk of cervical cancer in women aged less than 45 years using data from a case-control study conducted in Italy. Cases were 261 women aged < 45 years with histologically confirmed invasive cervical cancer. Controls were 257 women aged < 45 years, with acute, non-neoplastic conditions, judged to be unrelated to any of the known or suspected risk factors for cervical cancer. In comparison with women reporting one or no sexual partner, the multivariate odds ratio (OR) of cervical cancer was 2.4 (95% confidence interval, CI, 1.3-4.6), for women reporting two or more sexual partners, and, in comparison with women reporting their first intercourse at 17 years of age or before, the multivariate OR was 0.5 (95% CI 0.3-0.9) in women aged > or =23 years at first intercourse. The risk of cervical cancer was higher in parous women and increased with number of births (OR = 8.1 for three or more births). Among parous women the risk tended to increase with later age at last birth; in comparison with parous women reporting their last birth before age 25, the OR was 1.9 in those reporting their last birth at > or =35 years. No clear association emerged between oral contraceptive use, smoking, education, social class and risk of cervical cancer.


*British Journal of Cancer* (1998) **77**(5), 838–841
© 1998 Cancer Research Campaign

### Determinants of risk of invasive cervical cancer in young women

**F Parazzini[1,2], L Chatenoud[1], C La Vecchia[1,3], E Negri[1], S Franceschi[4] and G Bolis[2,5]**

[1]Istituto di Ricerche Farmacologiche Mario Negri, via Eritrea 62, 20157 Milan; [2]I Clinica Ostetrico Ginecologica, Università degli Studi di Milano, Milan; [3]Istituto di Statistica Medica e Biometria, Università degli Studi di Milano, via Venezian 1, 20133 Milan; [4]Centro di Riferimento Oncologico, Via Pedemontana Occidentale 33081 Aviano (PN); [5]Divisione di Oncologia Ginecologica, Istituto Nazionale Tumori, 20153 Milan, Italy

**Summary** We analysed determinants of risk of cervical cancer in women aged less than 45 years using data from a case–control study conducted in Italy. Cases were 261 women aged < 45 years with histologically confirmed invasive cervical cancer. Controls were 257 women aged < 45 years, with acute, non-neoplastic conditions, judged to be unrelated to any of the known or suspected risk factors for cervical cancer. In comparison with women reporting one or no sexual partner, the multivariate odds ratio (OR) of cervical cancer was 2.4 (95% confidence interval, CI, 1.3–4.6), for women reporting two or more sexual partners, and, in comparison with women reporting their first intercourse at 17 years of age or before, the multivariate OR was 0.5 (95% CI 0.3–0.9) in women aged ≥ 23 years at first intercourse. The risk of cervical cancer was higher in parous women and increased with number of births (OR = 8.1 for three or more births). Among parous women the risk tended to increase with later age at last birth; in comparison with parous women reporting their last birth before age 25, the OR was 1.9 in those reporting their last birth at ≥ 35 years. No clear association emerged between oral contraceptive use, smoking, education, social class and risk of cervical cancer.

**Keywords:** cervical cancer; risk factors; reproductive factor; oral contraceptives

Over the last few decades, cervical cancer mortality has declined in several countries; this, together with decreasing gastric cancer mortality, has been the main determinant of the favourable pattern in cancer mortality for young and middle-aged women (Devesa et al, 1987; 1995; Cuzick and Boyle, 1988; Decarli et al, 1993; Beral et al, 1994). These downward trends, however, generally started flattening off in younger women in the early 1970s, and in some countries (for example Britain) cervical cancer incidence and mortality in younger women have been rising again in more recent years (Cook and Draper, 1984; Cuzick and Boyle, 1988).

This has been generally attributed to changes in sexual habits in the younger generations, but epidemiological data on determinants and characteristics of cervical cancer in young women are scanty. In a recent case–control study conducted in the Greater London area in women under the age of 40 years, only factors related to sexual behaviour were found to be associated with cervical cancer risk, but no significant association emerged with parity or oral contraceptive use (Cuzick et al, 1996), which have been associated with the risk of cervical cancer in elderly women (WHO Collaborative Study of Neoplasia and Steroid Contraceptives, 1993). A clear definition of similarities and differences in the epidemiological characteristics of cervical cancer in young and elderly women may help understand potentially different effects of various mechanisms (viral, genetic or hormonal) in cervical carcinogenesis.

Thus, we analysed determinants of risk of cervical cancer diagnosed in women aged < 45 years, using data from a large case–control study on risk factors for cervical cancer conducted in Italy.

*Received 11 June 1997*
*Accepted 10 October 1997*

*Correspondence to:* Fabio Parazzini

### SUBJECTS AND METHODS

Data were derived from a hospital-based case–control study of invasive cervical cancer conducted between 1981 and 1993 in the greater Milan area (Parazzini et al, 1989). This study included 796 histologically confirmed cases of invasive cervical cancer and 919 controls younger than 75 years. In the present paper, we have considered only subjects aged < 45 years at diagnosis.

Cases were 261 women (median age 38 years, range 22–44 years) with histologically confirmed invasive cervical cancer admitted to the Obstetrics and Gynecology Clinics of the University of Milan, the National Cancer Institute and the Ospedale Maggiore of Milan (which includes the four largest hospitals in Milan).

The comparison group consisted of women aged less than 45 years, with acute conditions judged to be unrelated to any of the known or suspected risk factors for cervical cancer, who had been admitted to the same network of hospitals where the cases had been identified (chiefly the Ospedale Maggiore of Milan and several specialized university clinics), and were from similar catchment areas as cases. Women were not included if they were admitted for gynaecological, hormonal or neoplastic diseases, or had undergone total hysterectomy. A total of 257 controls were interviewed (median age 37, range 16–44 years). Of these 32% were admitted for traumatic conditions (mostly fractures and sprains), 30% had non-traumatic orthopaedic disorders (mostly lower back pain and disc disorders), 15% surgical conditions (mostly abdominal, such as acute appendicitis or strangulated hernia), and 22% had other illnesses, such as ear, nose and throat

**Table 1** Distribution of 261 cervical cancer cases and 257 controls according to age and selected factors, Italy, 1981–93

| | Cases Number (%) | Controls Number (%) | OR (95% CI) | |
|---|---|---|---|---|
| | | | MH | MLV |
| Age (years) | | | | |
| ≤ 30 | 26 (10.0) | 44 (17.1) | – | – |
| 31–35 | 38 (14.6) | 45 (17.5) | – | – |
| 36–40 | 87 (33.3) | 81 (31.5) | – | – |
| 41–44 | 110 (42.1) | 87 (33.9) | – | – |
| Education (years) | | | | |
| <7 | 136 (52.1) | 94 (36.6) | 1+ | 1+ |
| 7–11 | 65 (24.9) | 91 (35.4) | 0.6 (0.4–0.8) | 0.6 (0.4–1.0) |
| ≥ 12 | 60 (23.0) | 72 (28.0) | 0.6 (0.4–1.0) | 0.8 (0.4–1.2) |
| $\chi^2_1$ trend | | | 5.6 $P = 0.02$ | 2.9 $P = 0.09$ |
| Social class | | | | |
| I–II | 23 (8.8) | 25 (9.7) | 1+ | 1+ |
| III | 110 (42.1) | 117 (45.5) | 1.0 (0.5–1.9) | 0.9 (0.4–1.7) |
| IV | 116 (44.4) | 102 (39.7) | 1.3 (0.7–2.4) | 1.1 (0.5–2.1) |
| Unknown | 12 (4.6) | 13 (5.1) | – | – |
| Number of sexual partners | | | | |
| 0–1 | 161 (61.7) | 185 (72.0) | 1+ | 1+ |
| 2–3 | 70 (26.8) | 43 (16.7) | 1.9 (1.2–3.0) | 2.4 (1.5–3.8) |
| ≥ 4 | 30 (11.5) | 29 (11.3) | 1.3 (0.7–2.3) | 2.4 (1.3–4.6) |
| $\chi^2_1$ trend | | | 4.0 $P < 0.05$ | 13.6 $P < 0.001$ |
| Age at first intercourse (years) | | | | |
| ≤ 17 | 84 (32.2) | 65 (25.3) | 1+ | 1+ |
| 18–20 | 108 (41.4) | 102 (39.7) | 0.7 (0.4–1.1) | 0.7 (0.4–1.1) |
| 21–22 | 26 (10.0) | 27 (10.5) | 0.6 (0.3–1.1) | 0.6 (0.3–1.3) |
| ≥ 23 | 43 (16.5) | 63 (24.5) | 0.4 (0.2–0.7) | 0.5 (0.3–0.9) |
| $\chi^2_1$ trend | | | 10.5 $P = 0.001$ | 4.9 $P = 0.03$ |
| Smoking habits | | | | |
| Never smoked | 139 (53.3) | 142 (55.3) | 1+ | 1+ |
| Ex-smoker | 18 (6.8) | 17 (6.6) | 1.1 (0.5–2.2) | 1.3 (0.6–2.8) |
| Current smoker | 104 (39.5) | 98 (38.1) | 1.1 (0.8–1.6) | 1.1 (0.8–1.7) |
| Number of cigarettes per day* | | | | |
| < 5 | 12 (11.5) | 14 (14.2) | 0.8 (0.4–1.8) | 0.9 (0.4–2.3) |
| 5–14 | 45 (43.3) | 35 (35.7) | 1.4 (0.8–2.4) | 1.6 (0.8–2.9) |
| ≥ 15 | 47 (45.2) | 49 (56.0) | 1.0 (0.6–1.6) | 0.9 (0.5–1.5) |

+Reference category; OR, odds ratio; CI, confidence interval; MH, Mantel–Haenszel estimates adjusted for age; MLV, multivariate estimates including terms for age, education (except for social class estimates), calendar year at interview, parity, number of sexual partners and oral contraceptive use. *Current smokers only.

or dental disorders. Less than 2% of eligible women (cases and controls) refused to be interviewed.

The structured questionnaire included information on personal characteristics and habits, education and other socioeconomic factors, general lifestyle habits, such as smoking, alcohol and coffee consumption, a few indicators of gynaecological and obstetric history, related medical history, history of lifetime use of oral contraceptives, hormone replacement therapy in menopause and female hormone preparations for other indications.

### Data analysis

Odds ratios (ORs) of cervical cancer, and the corresponding 95% confidence intervals (CIs), were first computed with allowance for age (Mantel and Haenszel, 1959). They were then derived using unconditional multiple logistic regression, fitted by the method of maximum likelihood (Baker and Nelder, 1978), including terms for age in quinquennia, calendar year at interview, education, parity, number of sexual partners, oral contraceptive use and lifetime number of Pap smears.

### RESULTS

The distribution of cases and controls according to age, education, indicators of sexual habits and smoking is shown in Table 1. Cases tended to be less educated: in comparison with women reporting < 7 years of schooling, the multivariate OR estimates were 0.6 and 0.8 respectively for women reporting 7–11 and ≥ 12 years of schooling, but the trend in risk was not significant. No association emerged between social class and risk of cervical cancer.

The risk of cervical cancer increased with lifetime number of sexual partners, and decreased with increasing age at first intercourse. In comparison with women reporting one or no sexual partners, the multivariate OR of cervical cancer was 2.4 for women reporting two or more sexual partners and, in comparison with women reporting their first intercourse at 17 years of age or before, the multivariate OR was 0.5 in women aged ≥ 23 years at first intercourse. No significant association emerged between smoking and risk of cervical cancer.

Contraceptive habits are considered in Table 2. No significant relationship emerged between oral contraceptive use and risk of

© Cancer Research Campaign 1998

**Table 2** Distribution of 261 cervical cancer cases and 257 controls according to selected contraceptive habits. Italy 1981–93.

| | Cases Number (%) | Controls Number (%) | OR (95% CI) MH | OR (95% CI) MLV |
|---|---|---|---|---|
| Oral contraceptive use | | | | |
| Never | 165 (63.2) | 172 (66.9) | 1+ | 1+ |
| Ever | 96 (36.8) | 85 (33.1) | 1.2 (0.9–1.8) | 1.0 (0.7–1.6) |
| Time since last oral contraceptive use | | | | |
| Never | | | 1+ | 1+ |
| Current | 17 (6.5) | 22 (8.6) | 1.0 (0.5–2.1) | 1.0 (0.5–2.1) |
| <10 years | 64 (24.5) | 44 (17.1) | 1.6 (1.0–2.6) | 1.6 (1.0–2.5) |
| ≥ 10 years | 15 (5.7) | 19 (7.4) | 0.7 (0.4–1.5) | 0.6 (0.3–1.3) |
| Intrauterine device use | | | | |
| Never | 238 (91.2) | 223 (86.8) | 1+ | 1+ |
| Ever | 23 (8.8) | 34 (13.2) | 0.6 (0.3–1.0) | 0.4 (0.2–0.7) |

+Reference category; OR, odds ratio; CI, confidence interval; MH, Mantel–Haenszel estimates adjusted for age; MLV, multivariate estimates including terms for age, education, calendar year at interview, parity, number of sexual partners, oral contraceptive use and lifetime number of Pap smears.

**Table 3** Distribution of 261 cervical cancer cases and 257 controls according to reproductive factors. Italy, 1981–93

| | Cases Number (%) | Controls Number (%) | OR (95% CI) MH | OR (95% CI) MLV |
|---|---|---|---|---|
| Parity | | | | |
| 0 | 28 (10.7) | 77 (30.0) | 1+ | 1+ |
| 1 | 56 (21.5) | 65 (25.3) | 2.2 (1.2–4.0) | 2.4 (1.3–4.5) |
| 2 | 90 (34.5) | 77 (30.0) | 2.9 (1.7–5.2) | 3.8 (2.0–7.0) |
| ≥3 | 87 (33.3) | 38 (14.8) | 5.8 (3.1–10.7) | 8.1 (4.1–16.2) |
| $\chi^2_1$ trend | | | 31.2 $P < 0.0001$ | 36.8 $P < 0.0001$ |
| Age at first birth | | | | |
| ≤19 | 54 (23.2) | 25 (13.9) | 1+ | 1+ |
| 20–24 | 105 (45.1) | 82 (45.6) | 0.6 (0.3–1.0) | 0.6 (0.4–1.2) |
| 25–29 | 55 (23.6) | 55 (30.6) | 0.4 (0.2–0.8) | 0.6 (0.4–1.2) |
| ≥30 | 19 (8.2) | 18 (13.9) | 0.4 (0.2–1.0) | 0.5 (0.2–1.3) |
| $\chi^2_1$trend | | | 5.9 $P = 0.0155$ | 2.5 $P = 0.116$ |
| Age at last birth | | | | |
| ≤24 | 66 (28.3) | 51 (28.3) | 1+ | 1+ |
| 25–29 | 84 (36.1) | 75 (41.7) | 0.9 (0.5–1.7) | 0.9 (0.5–1.5) |
| 30–34 | 56 (4.0) | 44 (24.4) | 1.0 (0.6–1.7) | 0.9 (0.5–1.7) |
| ≥35 | 27 (11.6) | 10 (5.6) | 2.2 (0.9–5.1) | 1.9 (0.8–4.5) |
| $\chi^2_1$trend | | | 1.8 (NS) | 0.8 (NS) |
| Spontaneous abortions | | | | |
| 0 | 195 (74.7) | 212 (82.5) | 1+ | 1+ |
| ≥1 | 66 (25.3) | 45 (17.5) | 1.5 (1.0–2.3) | 1.3 (0.8–2.1) |
| Induced abortions | | | | |
| 0 | 189 (72.4) | 203 (79.0) | 1+ | 1+ |
| ≥1 | 72 (27.6) | 54 (21.0) | 1.3 (0.9–2.0) | 1.0 (0.6–1.5) |

+Reference category; OR, odds ratio; CI, confidence interval; MH, Mantel–Haenszel estimates adjusted for age; MLV, multivariate estimates including terms for age, education, calendar year at interview, parity, number of sexual partners, oral contraceptive use and lifetime number of Pap smears. NS, not significant.

cervical cancer. However, in comparison with never users, women reporting OC use in the 10 years before interview were at a higher risk (OR 1.6, 95% CI 1.0–2.5).

The relationship between reproductive history and risk of cervical cancer is considered in Table 3. The risk of cervical cancer was higher in parous women and increased with number of births (to 8.1 for women with three or more births). We have also analysed the relationship between parity and risk of cervical cancer in strata of age. In comparison with nulliparae, the ORs of cervical cancer were, respectively, for women aged < 35 years and 35–44 years: 3.7 (95% CI 1.5–8.8) and 1.5 (95% CI 0.6–3.8) for women reporting one birth; and 6.7 (95% CI 2.7–16.3) and 2.9 (95% CI 1.3–6.8) for those reporting two or more births. Considering parous women only, in comparison with women reporting their first birth at 19 years of age or before, the risk of cervical cancer tended to be lower in women reporting their first birth at 20 or more, but the ORs and the trend in risk were not significant. The risk tended to increase with age at last birth; in comparison with parous women

© Cancer Research Campaign 1998

reporting their last birth below age 25, the multivariate OR, was 1.9 (95% CI 0.8–4.5) in those reporting their last birth at ≥ 35 years of age. No clear relationship emerged between cervical cancer risk and number of spontaneous or induced abortions.

### DISCUSSION

This study confirms that invasive cervical cancers in young women share several risk factors with the disease in elderly women. Thus, the risk of invasive cervical cancer in women aged < 45 years was higher in women reporting multiple sexual partners and in multiparae. No significant association emerged with contraceptive methods and smoking, although the upper confidence limit of ORs for these two factors was close to 2. Less educated women tended to be at higher risk, but the multivariate trend in risk was not significant.

Potential limitations of this study should be considered as this was a hospital-based case–control study with all the consequent implications. However, particularly in consideration of the rather sensitive nature of the interview, the similar interview setting for cases and controls should have helped reduce potential information bias. Young women admitted to hospital for traumatic conditions may differ in their general lifestyle habits from the general population. However, no difference emerged in the ORs when the analysis considered the three main categories of controls separately. Selection should not represent a major problem in this study as cases and controls were identified in institutions covering broadly similar catchment areas, and participation was almost complete. Likewise, information bias can hardly have had a role on variables such as parity or age at pregnancy. In relation to confounding, simultaneous allowance for several potential distorting factors, including measures of social status and indicators of sexual habits, did not appreciably modify the associations observed.

A case–control study conducted in London, including 121 women aged < 40 years, found a strong association between number of sexual partners and the risk of cervical cancer (Cuzick et al, 1996). Those findings and the present results confirm the role of sexual habits (which are a likely indicator of infectious factors) in the carcinogenic process of cervical neoplasia (La Vecchia et al, 1986; Munoz et al, 1992). In contrast with the study by Cuzick et al (1996), we found a relationship between parity and risk of the disease of similar magnitude, or even stronger, than previously reported from the overall dataset in this population (Parazzini et al, 1989). Parity, besides being a marker of sexual activity, may act through a hormone-mediated mechanism on one of the later stages of the carcinogenic process, and therefore be more evident at a younger age (Parazzini et al, 1997). However, parity is a non-specific marker of hormonal factors.

Low education and low social class are recognized risk factors for cervical cancer (Brinton, 1992), but no association emerged in this study. Likewise, no relationship emerged between cervical cancer in young women and education and social class in a British study (Cuzick et al, 1996). Although the interpretation is still open to debate, this has been related to a cohort effect in sexual behaviour and exposure to other cervical cancer risk factors in various social classes.

Smoking is another risk factor for cervical cancer (Winkelstein, 1990), but in this and in a previous study (Cuzick et al, 1996) it was not related with the risk of invasive cervical cancer in young women. This finding, if not due to chance, is not easy to explain. The cervical epithelium of smokers contains fewer Langerhans' cells than that of non-smokers (Barton et al, 1988), thus favouring viral lesions. This facilitating effect may be more important for less aggressive viral strains, and cervical cancer in young women may therefore be more frequently related to more aggressive viral strains. Otherwise, tobacco smoking, by favouring viral lesions, may well act on one of the first stages of the process of carcinogenesis, and therefore require a long time to have a measurable impact on cervical cancer risk.

### ACKNOWLEDGEMENT

This work was conducted within the framework of the CNR (Italian National Research Council) Applied Projects 'Clinical Applications of Oncological Research' (Contract No. 96.00759.PF39, No. 96.00701.PF39), and 'Risk Factors for Diseases' (Contract No. 95.00952.PF41), and with the contribution of the Italian Association for Cancer Research. The authors wish to thank Ms Ivana Garimoldi for editorial assistance.

### REFERENCES

Baker NJ and Nelder JA (1978) *The GLIM System, Release 3*. Numerical Algorithms Group: Oxford

Barton SE, Maddox PH, Jenkins D, Edwards R, Cuzick J and Singer A (1988) Effect of cigarette smoking on cervical epithelial immunity: a mechanism for neoplastic change? *Lancet* **ii:** 652–654

Beral V, Hermon C and Munoz N (1994) Cervical cancer. *Cancer Surv* **19/20:** 265–685

Brinton LA (1992) Epidemiology of cervical cancer – overview. In *The Epidemiology of Cervical Cancer and Human Papillomavirus*, Munoz N, Bosch FX, Shah KV and Meheus A (eds), pp. 3–23 International Agency for Research on Cancer IARC: Lyon

Cook GA and Draper GJ (1984) Trends in cervical cancer and carcinoma in situ in Great Britain. *Br J Cancer* **50:** 367–375

Cuzick J and Boyle P (1988) Trends in cervix cancer mortality. *Cancer Surv* **7:** 417–439

Cuzick J, Sasieni P and Singer A (1996) Risk factors for invasive cervix cancer in young women. *Eur J Cancer* **32A:** 836–841

Decarli A, La Vecchia C, Negri E and Cislaghi C (1993) Cancer mortality in Italy, 1989, and an overview of trends from 1955 to 1989. *Tumori* **79:** 151–165

Devesa SS, Silverman DT, Young JL, Pollack ES, Brown CC, Horm JW, Percy CL, Myers MH, McKay FW and Fraumeni JF (1987) Cancer incidence and mortality trends among whites in the United States, 1947–1984. *J Natl Cancer Inst* **79:** 701–770

Devesa SS, Blot WJ, Stone BJ, Miller BA, Tarone RE and Fraumeni JF (1995) Recent cancer trends in the United States. *J Natl Cancer Inst* **87:** 175–782

La Vecchia C, Franceschi S, Decarli A, Fasoli M, Gentile A, Parazzini F and Regallo M (1986) Sexual factors, venereal diseases, and the risk of intraepithelial and invasive cervical neoplasia. *Cancer* **58:** 935–941

Mantel N and Haenszel W (1959) Statistical aspects of the analysis of data from retrospective studies of disease. *J Natl Cancer Inst* **22:** 719–748

Muñoz N, Bosch FX, De Sanjose S, Tafur L, Izarzugaza I, Gili M, Viladiu P, Navarro C, Martos C, Ascunce N, Gonzalez LC, Kaldor JM, Guerrero E, Lorincz A, Santamaria M, Alonso De Ruiz P, Aristizabal N and Shah K (1992) The causal link between human papillomavirus and invasive cervical cancer: a population-based case-control study in Colombia and Spain. *Int J Cancer* **52:** 743–749

Parazzini F, La Vecchia C, Negri E, Cecchetti G and Fedele L (1989) Reproductive factors and the risk of invasive and intraepithelial cervical neoplasia. *Br J Cancer* **59:** 805–809

Parazzini F, Franceschi S, La Vecchia C, Chatenoud L and Di Cintio E (1997) The epidemiology of female genital tract cancers. *Int J Gynecol Cancer* **7:** 169–181

WHO Collaborative Study of Neoplasia and Steroid Contraceptives (1993) Invasive squamous-cell cervical carcinoma and combined oral contraceptives: results from a multinational study. *Int J Cancer* **55:** 228–236

Winkelstein W JR (1990) Smoking and cervical cancer – Current status: A review. *Am J Epidemiol* **131:** 945–957

© Cancer Research Campaign 1998